# Management of Hepatic Encephalopathy by Traditional Chinese Medicine

**DOI:** 10.1155/2012/835686

**Published:** 2012-03-05

**Authors:** Chun Yao, Nong Tang, Guoxiang Xie, Xiaojiao Zheng, Ping Liu, Lei Fu, Wu Xie, Fan Yao, Houkai Li, Wei Jia

**Affiliations:** ^1^Guangxi College of Traditional Chinese Medicine, Nanning, Guangxi 530001, China; ^2^Department of Nutrition, University of North Carolina at Greensboro, North Carolina Research Campus, Kannapolis, NC 28081, USA; ^3^Institute of Liver Diseases, Shanghai University of Traditional Chinese Medicine, Shanghai 201203, China

## Abstract

In spite of the impressive progress in the investigation of hepatic encephalopathy (HE), the complex mechanisms underlying the onset and deterioration of HE are still not fully understood. Currently, none of the existing theories provide conclusive explanations on the symptoms that link liver dysfunction to nervous system disorders and clinical manifestations. This paper summarized the diagnostic and therapeutic approaches used for HE in modern medicine and traditional Chinese medicine and provided future perspective in HE therapies from the viewpoint of holistic and personalized Chinese medicine.

## 1. Introduction

Hepatic encephalopathy (HE), also known as portosystemic encephalopathy, is defined as a spectrum of neuropsychiatric abnormalities in patients with liver dysfunction, after exclusion of other known brain diseases [1–3]. The aggravation of HE will result in hepatic coma or coma hepaticum, which may ultimately lead to death [4]. It is believed that the increase of harmful substances entering brain from blood is the main cause of HE, and currently the identified causal factors for HE are ammonia [5, 6], *γ*-aminobutyric acid (GABA) [7, 8], false neurotransmitters [9, 10], and the imbalance of certain amino acids in plasma [11, 12]. In spite of the impressive progress in research aiming to uncover the etiology of HE, the complex mechanisms underlying the onset and deterioration of HE and related conditions are still not fully understood. Currently, none of the existing theories provide conclusive explanations on the symptoms that link liver dysfunction to nervous system disorders and clinical manifestations. Generally, it has been accepted that high blood ammonia, which is not properly metabolized in and removed from liver because of the hepatic dysfunction, is closely associated with dysregulation of central nervous system (CNS). The elevation of neurotoxins such as ammonia in blood and CNS impairs the related neurotransmitter system and leads to the functional disorder of CNS. Such a complicated pathology of HE implies the possibility of systematic involvement of multiple organs in orchestrating the development of HE. Therefore, it is necessary to adopt a systems strategy with interdisciplinary studies to understand how dysregulated metabolites disturb the organ-organ (liver-brain) interactions and eventually to uncover the mechanisms of HE at a systems level.

Traditional Chinese medicine (TCM) typically involves a personalized diagnosis and the use of herbal formulae of between 10–20 separate herbal ingredients selected from material medica of several thousand herbs that are prepared either as a boiled decoction, as dried herbal extracts, or taken as pills [13]. Diagnostic and therapeutic treatment principles are framed according to the TCM understanding of pathological processes. A good practice of TCM is usually considered to require a TCM pattern identification based on clinical manifestation followed by the use of individualized herbal decoctions that are adapted to address the particular TCM pattern of each patient [14]. Since the treatment will change following the changing TCM patterns and clinical manifestations. TCM is a dynamic and highly responsive system of medicine that resonates strongly with the increasing emphasis within systems biology strategy for the use of both multiple approaches to achieve optimum diagnosis and individualized treatments to take into account variable responses to modern drugs.

In the theory of TCM, the onset of HE is due to the invasion of damp and heat in triple burners which leads to phlegm and stagnation of Qi, which eventually causes the disability of thinking in HE patients. Such a traditional theory appears to be consistent with the modern theories of HE etiology. Given the holistic and personalized nature of TCM, HE and its clinical manifestations are divided into various TCM patterns (phenotypes) with different characteristics, which necessitate different therapeutic methods in TCM. In this paper, we summarized the theories and therapeutic methods of HE both in modern medicine and TCM and provided future perspective in HE therapies from the viewpoint of systems biology.

## 2. The Diagnosis of HE in Modern Medicine

To date, there are no gold-standard diagnostic procedures for HE with high sensitivity and specificity in modern medicine. HE patients usually have advanced chronic liver disease and thus have many of the physical and laboratory stigmata associated with severe hepatic dysfunction. Physical features may include muscle wasting, jaundice, ascites, palmar erythema, edema, spider telangiectasias, and fetor hepaticus [15, 16]. However, some of these features (such as muscle wasting, spider telangiectasias, and palmar erythema) are usually absent in HE patients with fulminant hepatic failure who are previously healthy, because the development of these features requires a relatively longer period of hepatic dysfunction. As a result, substantial technical and laboratory examinations are needed for diagnosis of HE patients, which include psychological test, electrophysiological test, and imaging.

### 2.1. Psychological Test

The most common tests used in clinics are number connection test (NCT), line tracking test (LTT), serial dotting test (SDT), continuous reaction time (CRT), critical frequency scintillometer (CFS), and wisconsin Ccard sorting test (WCST). The combination of these tests will increase the accuracy of HE determination, avoiding false diagnosis based on a single test. For example, HE psychological testing, a combined test group including NCT, DST, LTT and SDT, has become a rapid and practical procedure that takes less than 20 minutes and achieves 96% sensitivity and 100% specificity.

### 2.2. Electrophysiological Test

Electrophysiological and electropsychological changes can be tested by electroencephalogram (EEG) and brain electrical activity mapping (BEAM). EEG can be used not only as an evaluation tool but for early diagnosis as well. However, it may be difficult to evaluate the disease objectively as it lacks specificity [17]. Recently, based on the EEG assessment, a test called artificial neural network-expert system (ANNES) with the computer analysis technology is proposed as an expert system to overcome this problem [18]. Additionally, analysis of the EEG utilizing a spatiotemporal decomposition technique (SEDACA) provides significantly more diagnostic information on the neuropsychiatric status of HE patients than obtained conventionally [19].

### 2.3. Imaging

The imaging methods for HE include computerized tomography (CT) and magnetic resonance imaging (MRI), magnetic resonance spectroscopy (MRS), single photon emission computed tomography (SPECT), and positron emission tomography (PET) [20]. CT and MRI are mainly used to detect brain morphology, such as hydrocephalus in the acute HE patients and encephalatrophy, especially in lobus frontalis in the chronic HE patients [21]. MRS is mainly used to detect the changes of compounds in cells, analyzing the concentrations of such metabolites as glycoconjugates, amino acids, cholines, phospholipids, and creatine to help early diagnosis. Both SPECT and PET are used extensively to assess brain perfusion, which is typically less “active” in HE patients than in healthy people.

## 3. Therapies of HE in Modern Medicine

The treatments for HE typically include (1) elimination or correction of the underlying factors participating in HE, (2) restoring metabolic homeostasis, (3) promoting regeneration of liver cells, (4) antibiotic agents that inhibit mucosal glutaminase in the intestine to reduce ammonia production in the gut, and (5) artificial liver support or liver transplantation [22–26]. Most patients show clinical signs of improvement in the symptoms of HE within 24–48 hours of initiation of treatment. Serum levels of ammonia might lag behind the clinical response.

### 3.1. Dietary Regulation of Homeostasis

Restriction of protein intake in diet is preferred for HE therapy. It is advised to consume more calories from vegetable and dairy protein, because vegetable protein is rich in branched-chain amino acids and nonabsorbent fiber, which are beneficial for the balance of normal gut microbiota and acidifying the intestinal tract. It is also necessary to uptake sufficient carbohydrate and vitamins, that is, vitamin C can reduce the level of pH in blood and divert ammonia from brain to blood [27–29]. On the other hand, drinking sufficient water is helpful for maintaining homeostasis of the body, which protects body from hypokalemia, hyperkalemia, hyponatremia, hypocalcemia, hypomagnesemia, and metabolic alkalosis. Additionally, some other ways are available for keeping the homeostasis such as plasma or albumin transfusion, to increase plasma colloid osmotic pressure, improve hypoxemia and hydrocephalus, and prevent hemorrhage and bacterial infection.

### 3.2. Elimination of Blood Ammonia

Elimination of blood ammonia is critical for HE treatment. Lactulose is widely used as a standard medicine to evaluate the effect of new drug for HE [30]. Besides, Lactitol is also well practiced for HE therapy with comparable effect to lactulose, but with better tolerance [31, 32]. Although Neomycin is effective for HE patients, long-term usage is prohibited for its toxicity [33, 34]. Oral administration of L-7-ornithine-aspartate (OA) can effectively eliminate the level of blood ammonia [35]. Recent studies show that the concentration of blood ammonia in HE patients was significantly decreased by Rifaximin, as well as amelioration of the patients' condition [36–38].

### 3.3. Supplementation of Branched-Chain Amino Acids

The administration of branched-chain amino acids (BCAAs) may help adjust the abnormal ratio of BCAAs to aromatic amino acids (AAAs) crossing the blood-brain barrier (BBB), so that the symptom of HE can be improved. A recent meta-analysis has shown that patients with cirrhosis who receive BCAAs are more likely to recover from HE than those who do not receive this supplement [39]. BCAAs improve levels of serum albumin, increase progression-free survival, and reduce both the number of hospitalizations and the length of hospital stays in patients with cirrhosis [40]. These amino acids can be administered orally as well as intravenously.

### 3.4. Use of False Neurotransmitter Antagonist

According to the theory of false neurotransmitter for HE development [2, 10], antagonists of false neurotransmitter could be used for HE therapy, including bendopa, dopamine agonist Bromocriptine, and opium receptor Narcon. Bendopa could pass the BBB and flow into brain tissue and produce dopamine and norepinephrine by enzymatic catalysis, which are substitutes for the false neurotransmitter and help to recover nerve function. Bromocriptine could agitate postsynaptic dopamine receptor to upregulate prolactin with nerve transmitting function strengthened. Using Bromocriptine alone or with Lactulose together is especially effective to those chronic HE patients who are insensitive to Neomycin or Lactulose. Narcon can cross BBB easily and attenuate the inhibited effect on CNS caused by redundant opioid peptides. Clinical data showed that Narcon is helpful to improve the consciousness of HE patients. However, evidence has indicated that obvious variations exist in therapeutic effectiveness of these medicines on HE patients [41, 42].

## 4. Treatment of HE in TCM

In TCM, the phenotype of HE is the result of impaired resistance to damp and heat environment (two of the six exogenous pathogens in TCM including wind, cold, heat, damp, dryness, and fire), along with the reduced function of middle burner (the middle part of triple burners in TCM, referring mainly to the organs located between diaphragm and navel, including stomach and spleen) to excrete toxic substances. The accumulated toxic substances in the middle burner spread to the triple burners (including upper, middle, and lower burner, covering all of the organs) and affect the upper orifices (upper orifices are the openings on the face, such as the eyes, ears, nose, and mouth). The essential substances of the organs are distributed through these orifices, so any pathologic change of these orifices contributes to a diagnosis of the disorders of these organs. The pathogenesis in TCM view is consistent with the modern medicine in that abnormal accumulation of metabolites, especially the production, absorption, and distribution of endotoxin in the patients with liver failure will cause the metabolic imbalance in blood. As a result, the therapeutic strategy for HE under TCM includes purgation and eliminating stasis in organs and inducing resuscitation, which is holistic and dynamic in nature.

### 4.1. Treatment of HE according to TCM Patterns

TCM pattern differentiation is a method to analyze and characterize the clinical manifestations of a disease, a process in which the geographical location, nature, occurrence, and development of the diseased and pathogenic factors are taken into account. Once a specific pattern of an HE patient is identified, a specific treatment strategy will be used to correct or mitigate the pattern of the patient.  Table 1 provides a summary of typical TCM patterns of HE and their subsequent treatments.

#### 4.1.1. TCM Pattern of HE—Invasion of Pericardium by Excessive Heat Toxin

The main clinical manifestation of this pattern is characterized as follows. High fever appears at night, severe jaundice with clear yellowing of the body and deteriorating fast, either coma and unconsciousness or disturbed emotion even delirium, constipation inducing distension and ascites, hemorrhinia, hematemesis, hematochezia, bright red tongue substance with yellow and dry tongue coating, taut thready or taut rapid pulse. Treatment should be cleansing the heat toxin and inducing resuscitation. TCM prescriptions commonly used in clinic are Purple Snowy Powder [43], Qing Ying Liang Xue Tang and Cow-bezoar Bolus for Resurrection [44], Antipyretic and Antitoxic Decoction [45], Coptidis Decoction for Detoxification, combined with Rhubarb and Treasured Bolus [46], and a new prescription made with some of the herbs from the three prescriptions Herbae Artemisiae Capillariae Decoction, Antiphlogistic Decoction of Five Drugs, and Cornus Rhinoceri and Rehmannia Decoction [45].

#### 4.1.2. TCM Pattern of HE—Dampness and Phlegm Accumulation Causing Mental Confusion

The main clinical manifestation of this syndrome is characterized as follows. Apparent symptom of jaundice, dark complexion, coma with nausea and vomiting, abdominal distension, high fever at the same time, urine with yellow color and small amount, exhaustion, chest distress, abdominal flatulence, bitterness in the mouth, dark red tongue substance with white greasy or yellow greasy tongue coating, soft and rolling pulse or soft and thready pulse. Treatment should be clearing away dampness, dispelling Phlegm and inducing resuscitation. TCM prescriptions commonly used in clinics are Herbae Artemisiae Capillariae Decoction [47], Artemisiae Scopariae and Poriae Powder [48], Phlegm-removing decoction, combined with Rhubarb and Storax Pill [46], Jufang Zhibao Dan [47], Yin Chen Si Ling Decoction, and Changpu Yujin Decoction [45].

#### 4.1.3. TCM Pattern of HE—Yin Deficiency of Liver and Kidney Coupled with Yang Excess of Liver

The main clinical manifestation of this syndrome is characterized as follows. Swarthy complexion, thin shape, faintness, coma, distracted, jerking movements in the extremities, red and dry tongue substance with little tongue coating, taut thready or taut rapid pulse. Treatment should be nourishing liver and kidney, and expelling wind and heat. TCM prescriptions commonly used in clinic are Du Xiao Ke Li [49], Yiguan Decoction [50], Cornu Satgae Decoction, and Subphrenic Recesses [45].

#### 4.1.4. TCM Pattern of HE—Exhaustion of Yin and Yang Coupled with Disturbance in Spirit

The main clinical manifestation of this syndrome is characterized as follows. Dottiness, coma, pale complexion, cold extremities, carphology, syncope with convulsion, slow reaction, weak breath, diaphoresis, incontinence of urine and feces, pale tongue substance without tongue coating, feeble and impalpable pulse. Treatment should be supplementing Qi and nourishing Yin, reduce resuscitation, and recuperate depleted Yang. TCM prescriptions commonly used in clinic are Pulse-Activating Powder or Cornus Rhinoceri and Rehmanniae Decoction [51], Shen Fu Long Mu Tang, or Ginseng Decoction [45].

### 4.2. TCM Treatmen—Purging Organs and Opening Orifices

The pathogenesis of HE mostly includes the deficiency of liver and kidney, phlegm retention and blood stasis, failure of Yang and Yin to raise and fall, respectively, which could be regarded as the declining function in distributing nutrients to the organ and excretion out of the organ, leading to the symptoms like coma, convulsion, and mental confusion [52]. The TCM pattern of the excess phlegm and serum stasis with the deficiency in both Yin and Yang of Qi and blood affect the mental stability. A clinical TCM retrospective survey with a large number of HE patients (*n* = 1072) and a prospective survey with 133 HE subjects revealed that the main cause of liver failure is a combination of toxin, phlegm, and blood stasis entangled in the body along with dampness, heat, and pestilence invasion [53]. Therefore, the TCM treatment approaches involve removing toxin, expelling blood stasis, and eliminating phlegm have been applied in the clinical treatment of HE. Several representative clinical studies are described using this approach in the following.

*Rhubarb* (*Rhei Radix and Rhizoma*) is a potent herb with purging activity, which can relieve internal heat and promote blood circulation by removing blood stasis and normalizing gallbladder to cure jaundice [54–56]. Li and Ma [57] applied a decoction of a single medicinal plant, *Rhubarb*, through colon infusion in 30 HE patients. About 30 g of Rhubarb was prepared to decoction in a 200 mL of water as an enema. This decoction was administered 1-2 times daily for 10 days as a course of treatment. Six patients experienced “complete remedy” (CR, defined as reaching and maintaining a conscious and lucid state of mind for 3 weeks after dose), 18 patients experienced “partial remedy” (PR, significant improvement of the symptoms), while 6 patients had no effect. The total efficacy (CR + PR) was 80%.

Lv and Li [58] applied a TCM agent, Tongfu Xiere Decoction, containing *Rhubarb, Dandelion, Magnolia Bark, Citri Immaturus, and Fructus Mume*, to 64 HE patients, with an attempt to relieve internal heat, and cool, promote blood flow, and eliminate phlegm and freeing channels. The patients were divided into two groups, a control group in which all subjects received intravenous infusion of 40 mL of Qingkailing (a TCM drug) injection, 250 mL of BCAA or 10 g of Hepa Merz, once a day, and a TCM group in which Tongfu Xiere Decoction was applied in addition to the treatments in the control group. The decoction was prepared as an enema and administered through colon infusion at 250 mL a day. Therapeutic efficacy in TCM group reached 93.94% while the control group reached 80.65%.

A combined Narcon and *Rhubarb* therapy for 62 HE patients was conducted by Huang [59] recently. The patients were randomly divided into two groups, conventional therapy group (*N* = 24) with an integrated approach comprising antibiotics treatment, balancing electrolytes, amino acids, and pH in body fluid, and so forth, and the treatment group (*N* = 38), in which intravenous infusion of Narcon and colon infusion of Rhubarb decoction were applied in addition to the approach used in the conventional therapy group. Narcon was infused at a dose of 4 mg in 500 mL of 5% Glucose, at 0.3 mg/h. The decoction of 30 g of *Rhubarb* in 500 mL water was applied once a day. The results showed a significant improvement in effectiveness in the treatment group with 94.7% efficacy (defined as showing a conscious state <48 h after dose) compared to an efficacy of 66.7% in the conventional therapy group.

A similar clinical investigation was conducted using the decoction of *Rhubarb* as an enema to treat 60 HE patients at the First Hospital affiliated to Guangxi University of Traditional Chinese Medicine. The patients were randomly divided into two groups, conventional therapy group (*N* = 30) with an integrated approach, and the treatment group (*N* = 30) with colon infusion of a decoction of *Rhubarb* and *Mume Fructus* were applied in addition to the approach used in the conventional therapy group. The decoction of Rhubarb and *Fructus Mume* (30 g : 30 g in 100 mL water) was applied once a day. After a 3-day course of treatment, the total effective rate (defined as HE symptoms improved by one stage (0–4 stages) within 48 h after dose) in the treatment group was 83.33%, higher than that (56.67%) in conventional therapy group [60].

## 5. Summary and Prospect

### 5.1. Summary of Therapies for HE with Modern Medicine and TCM

The current diagnoses of HE are still based on experimentalism lacking accurate and objective evaluation of the pathology in modern medicine due to the complexity of the HE pathogenesis. Current treatment of HE is focused on a comprehensive management of disease symptoms and improvement of patients' quality of lives, with less satisfactory effectiveness in reversing the pathological course of HE. On the other hand, long-term exposure to therapeutic drugs also results in drug resistance and dependence. As a result, no universally effective treatment has been generated in modern medicine. However, the successful use of TCM therapeutic approaches over the past decades suggests that alternative approaches be taken into consideration for HE therapy with holistic and personalized views and a multi-level and multipathway adjustment strategy. For example, the treatment with the strategy of purging organs and removing blood stasis has been increasingly accepted for HE therapy in TCM. Nevertheless, more well-designed studies should be conducted to further evaluate the clinical efficacy of TCM approaches and elucidate the complicated mechanisms of TCM treatment for HE patients.

### 5.2. Prospective

The brain and liver are key targets for damage induced by dysregulated metabolites often associated with gut-generated signals. Thus the gut-liver-brain axis is crucial for coordinating homeostasis and health. Therefore, interdisciplinary studies of how dysregulated metabolites disturb the gut-liver-brain interactions will uncover novel mechanisms of HE, which are essential for understanding the pathogenesis at a systemic level. Such knowledge is the basis for development of effectively preventive and therapeutic strategies in most-at-risk populations.

Recent studies suggest that HE seems to be the result of the energy metabolism defects in brain, neurotransmitter abnormity, and mutation of the receptors on the membrane of neuron. Thus, the pathogenesis of HE might be a result of systematic dysfunctions in multiple organs. Metabolomics, an important element for systems biology with genomics, transcriptomics, and proteomics, has been increasingly applied in identifying and quantifying significantly altered metabolites in cell, tissue, organ, or organism, as the end products of biological processes reflecting pathological change of diseases or the effects of medicine to the body. Serum metabolite profiling with 1H-NMR has been implemented in patients with normal, cirrhosis, or minimal HE, in which substantial differentiated metabolites have been identified among different groups [61]. The application of metabolomics to the study of HE will help understand the pathogenesis and provide a new method for early diagnosis of this disease. Metabolomics may be an effective technique linking quantitative changes of metabolites to syndromes of TCM because the various syndromes of TCM may result from global metabolic imbalances in the patients. As a result, metabolomics can be applied as a holistic profiling tool to unveil the veil of TCM diagnosis and therapies of HE. Such a novel clinical approach coupled with TCM strategies is expected to make breakthrough discoveries in the areas of characterizing metabolic phenotypes of HE, developing diagnostic and treatment biomarkers, and identifying herbal medicines suitable for HE treatment.

## Figures and Tables

**Table 1:** The typing, prescription, and treatments for different syndromes of HE.

Prescription	Medicinal herb ingredients
Invasion of pericardium^a^ by excessive heat and toxin	

Zi Xue Pill	*Gypsum Fibrosum, Gypsum Rubrum, Magnetitum, Talcum, Bubali Cornu, Saigae Tataricae Cornu, Aucklandiae Radix, Aquilariae Lignum Resinatum, Cimicifugae Rhizoma, Glycyrrhizae Radix et Rhizoma, Caryophylli Flos, Natrii Sulfas, Moschus, Cinnabaris*
Qing Ying Liang Xue Decoction	*Bubali Cornu, Salviae Miltiorrhizae Radix et Rhizoma, Artemisiae Scopariae Herba,*
An Gong Niu Huang Pill	* Imperatae Rhizoma, Bergenia Herba, Paeoniae Radix Rubra, Rehmanniae Radix, Moutan Cortex, Gardeniae Fructus Praeparatus, Rhei Radix et Rhizoma*
Qing Wen Bai Du Oral Solution	*Bubali Cornu, Coptidis Rhizoma, Scutellariae Radix, Artemisiae Scopariae Herba, Lysimachiae Herba, Gypsum Fibrosum, Anemarrhenae Rhizoma, Platycodonis Radix, Rhei Radix et Rhizoma, Gardeniae Fructus, Smilacis Glabrae Rhizoma, Alismatis Rhizoma, Plantaginis Semen, Aurantii Fructus Immaturus, Forsythiae Fructus, Rehmanniae Radix, Lophatheri Herba, Scrophulariae Radix*
Huang Lian Decoction for Detoxification Plus Zhi Bao Pill	*Coptidis Rhizoma, Phellodendri Chinensis Cortex, Scutellariae Radix, Gardeniae Fructus, Rhei Radix et Rhizoma * *Bubali Cornu, Bovis Calculus, Eretmochelys imbricata, Ambrum, Cinnabaris, Realgar, Moschus, Benzoinum*
Yin Chen Hao Decoction	*Artemisiae Scopariae Herba, Gardeniae Fructus, Rhei Radix et Rhizoma*
Wu Wei Detoxification Oral Liquid	*Lonicerae Japonicae Flos, Taraxaci Herba, Violae Herba, Begonia Fimbristipula Herba, Eupolyphaga or Steleophaga*
Xi Jiao Di Huang Decoction	*Bubali Cornu, Rehmanniae Radix, Paeoniae Radix Rubra, Moutan Cortex, Arnebiae Radix*

Pattern of mental confusion by dampness and phlegm^b^ accumulation	

Yin Chen Wu Ling Dispersing agent	*Artemisiae Scopariae Herba, Polyporus, Alismatis Rhizoma, Atractylodis Macrocephalae Rhizoma, Poria, Cinnamomi Ramulus*
Phlegm-removing Decoction with Da Huang	*Arisaematis Rhizoma, Pinelliae Rhizoma, Aurantii Fructus Immaturus Poria, Citri Exocarpium Rubrum, Cinnabaris, Acori Tatarinowii Rhizoma, Atractylodis Macrocephalae Rhizoma, Caryophylli Flos, Aquilariae Lignum Resinatum, Santalum album, *
Su He Xiang Pill	*Olibanum, Piperis Longi Fructus, Bubali Cornu, Benzoinum, Aucklandiae Radix, Cyperi Rhizoma, GinsengRadix et Rhizoma, Bambusae Caulis In Taenias, Glycyrrhizae Radix et Rhizoma, Rhei Radix et Rhizoma, Styrax, Moschus, Borneolum Syntheticum*
Ju Fang Zhi Bao Pill	* Bubali Cornu, Bovis Calculus, Eretmochelys imbricata, Ambrum, Cinnabaris, Realgar, Moschus, Benzoinum*
Yin Chen Si Ling Decoction	*Artemisiae Scopariae Herba, Poria, Alismatis Rhizoma, Polyporus, Gardeniae Fructus*
Chang Pu Yu Jin Decoction	*Acori Tatarinowii Rhizoma, Curcumae Radix, Arisaema Cum Bile, Pinelliae Rhizoma, Magnoliae Officinalis Cortex, Myristicae Semen, Polygalae Radix, Forsythiae Fructus, Pogostemonis Herba*

Pattern of Yin^c^ deficiency of liver and kidney and Yang^c^ excess of Liver	

Yi Guan Decoction	*Rehmanniae Radix, Angelicae Sinensis Radix, Lycii Fructus, Glehniae Radix, Ophiopogonis Radix, Toosendan Fructus*
Ling Yang Jiao Decoction	*Saigae Tataricae Cornu, Testudinis Carapax Et Plastrum, Rehmanniae Radix, Ligustri Lucidi Fructus, Eclipse Prostrala Herba, Dendrobii Caulis, Margaritifera Concha, Moutan Cortex, Paeoniae Radix Rubra, Bupleuri Radix, Prunellae Spica, Chrysanthemi Flos, Haliotidis Concha, Carthami Flos, Persicae Semen, Angelicae Sinensis Radix, Chuanxiong Rhizoma, Trogopteri xanthipes stool, Cyperi Rhizoma, Corydalis Rhizoma, Artemisiae Scopariae Herba*

Pattern of exhaustion of Yin and Yang, and disturbance in spirit	

Pulse-Activating Powder	*Ginseng Radix Et Rhizoma, Ophiopogonis Radix, Schisandrae Chinensis Fructus*
Xi Jiao Di Huang Decoction	*Bubali Cornu, Rehmanniae Radix, Paeoniae Radix Alba or Paeoniae Radix Rubra, Moutan Cortex*
Shen Fu Long Mu Decoction	*Ginseng Radix Et RhizomaRubra, Aconiti Lateralis Radix Praeparata, Astragali Radix, Ostreae Concha, Corni Fructus, Polygonati Rhizoma, Rehmanniae Radix, Rehmanniae Radix Praeparata, Schisandrae Chinensis Fructus, Scrophulariae Radix, Ophiopogonis Radix, Adenophorae Radix*

^a^Pericardium refers to an anatomical membrane surrounding heart, and physiologically it protects the heart. When exogenous pathogens invade the heart, the pericardium is always the first to be attacked. Invasion of the pericardium by pathogenic heat gives rise to symptoms of mental disturbances such as coma and delirium in TCM.

^b^Phlegm is usually secreted by dysfunction of lung and spleen, and occasionally by the consumption of body fluids by fire and heat evils. A disharmony of body fluids can produce either external, visible phlegm, such as sputum secreted by the respiratory tract, or internal, invisible phlegm.

^c^The Yin-Yang theory believes that the normal life activities of the human body result from the harmonious relation of the unity of opposites between Yin and Yang. The imbalance between Yin and Yang is one of the basic pathogenesis of a disease. All the pathological changes can be summarized as excess or deficiency of Yin or Yang. To be more concrete, “Yang excess leads to heat syndrome while Yin excess causes cold syndrome”; “Yang deficiency results in cold syndrome while Yin deficiency causes heat syndromes”; “Yang deficiency affects Yin while Yin deficiency affects Yang.”
